# Fungi involved in rhinosinusitis in arid regions: insights from molecular identification and antifungal susceptibility

**DOI:** 10.1128/spectrum.01831-23

**Published:** 2023-09-29

**Authors:** Shaoqin Zhou, Mawahib A. I. Ismail, Jochem B. Buil, Aida Gabr, Paul E. Verweij, El-Sheikh Mahgoub, Sybren de Hoog, Yingqian Kang, Sarah A. Ahmed

**Affiliations:** 1 School of Public Health, the key Laboratory of Environmental Pollution Monitoring and Disease Control, Ministry of Education, School of Basic Medical Science, Guizhou Medical University, Guiyang, China; 2 Radboudumc-CWZ Centre of Expertise for Mycology, Nijmegen, the Netherlands; 3 Department of Medical Microbiology, Radboudumc, Nijmegen, the Netherlands; 4 Mycology Reference Laboratory, University of Khartoum, Khartoum, Sudan; 5 Foundation Atlas of Clinical Fungi, Hilversum, the Netherlands; Institut Pasteur, Paris, France

**Keywords:** Sudan, arid climate, fungal rhinosinusitis, *Aspergillus*, molecular characterization, antifungal susceptibility

## Abstract

Fungal rhinosinusitis (FRS) is a common problem worldwide, with an increasing burden in arid climate regions. *Aspergillus* species are the most common causative agents involved. In the present study, we investigated the prevalence, molecular characterization, and antifungal susceptibility of opportunists causing FRS in Sudan on the basis of strains collected over a period of 5 years. β-Tubulin and calmodulin sequencing were used for species identification, and antifungal susceptibility profiles were evaluated by the protocol of the European Committee on Antimicrobial Susceptibility Testing (EUCAST). Predominant species belonged to the *Aspergillus flavus* complex (*n* = 244), *A. terreus* complex (*n* = 16), *A. fumigatus* complex (*n* = 7), and other fungi (*n* = 17). Molecular identification of 94 strains of *Aspergillus* revealed the following species: *A. flavus* (*n* = 88), *A. terreus* (*n* = 1), *A. citrinoterreus* (*n* = 2), *A. fumigatus* (*n* = 1), *A. caespitosus* (*n* = 1), and *A. sydowii* (*n* = 1). Several *A. flavus* and an *A. fumigatus* isolates showed reduced susceptibility to azoles (minimum inhibitory concentrations above the clinical breakpoints or epidemiological cutoff values). Despite several mutations revealed in *cyp51A* of these isolates, none could be directly linked to azole resistance. Molecular identification of fungi causing FRS is useful to identify cryptic species and for epidemiologic studies.

**IMPORTANCE:**

Fungal rhinosinusitis (FRS) is a significant clinical problem in arid regions. This study provides new insights into the prevalence, etiology, and antifungal susceptibility of FRS pathogens in Sudan, where the disease burden is high. *Aspergillus* species, particularly the *A. flavus* complex, were identified as the primary FRS pathogens in the region, with some evidence of antifungal resistance. The molecular identification of fungal species causing FRS is useful for detecting antifungal resistance, identifying cryptic species, and characterizing the epidemiology of the disease. The emergence of Azole resistance *Aspergilli* in Sudan highlights the need for continued surveillance and appropriate use of antifungal agents. These findings have important implications for clinical management, public health policy, and future research on FRS. Publishing this study in Microbiology Spectrum would enable other researchers and clinicians to build on these findings, ultimately improving the diagnosis, treatment, and prevention of FRS.

## INTRODUCTION

Fungal rhinosinusitis (FRS) is an infection of the nasal sinus membrane caused by various species of fungi. This can result in obstruction of the cavities and subsequent infection or infiltration of nearby structures, such as the orbit and palate, and even the intracranial space. The disease is a global health concern with a growing burden in arid and tropical regions, predominantly in Asia, the Middle East, and Northern Africa (1, 2). Estimates suggest that there are 1.5 million cases of FRS in India per year and up to 392 per 100,000 in Turkey (3). However, data from other countries on this disease are scant.

The widely accepted classification of FRS as suggested by the International Society for Human and Animal Mycology (ISHAM) https://www.isham.org/ divides the disorder into invasive and non-invasive types, based on histopathological demonstration of tissue invasion by fungal elements (4). Fungi responsible for FRS are found ubiquitously in the environment with *Aspergillus* being a commonly encountered genus in tropical and arid climate regions. Additionally, melanized fungi such as *Bipolaris* and *Curvularia* have also been reported as causative agents of fungal sinusitis (5). Furthermore, some species of Mucorales, such as *Mucor*, *Rhizopus*, and *Lichtheimia*, are encountered in a more severe and rapid form of infection (6).

The classical diagnostic modalities for FRS include direct microscopy, histopathology, and culture, with a combination of culture and histopathology providing the most accurate diagnosis. Identification of the fungi involved in FRS is crucial for administering effective antifungal therapy (7). Both phenotypic and genotypic methods have been used for identification of clinical isolates (8). However, the diagnostic potential of phenotypic identification is limited. The approach is unable to distinguish closely related species, especially in *Aspergillus*. This genus comprises 27 sections, with causative agents of disease of humans and animals mostly found in sections *Fumigati*, *Flavi*, *Terrei*, *Nigri*, and *Nidulans* (9). Distinction of *Aspergillus* species within these sections is optimal with a polyphasic approach incorporating both morphological and molecular methods, as recommended by most experts (10). Although the rDNA internal transcribed spacer (ITS) is widely accepted as the official DNA barcode for fungi, its resolution at the species level in *Aspergillus* may not be sufficient (11). As an alternative, the protein coding gene calmodulin (*CaM*) is suggested for routine identification. Additionally, the β-tubulin (*BenA*) gene has been shown to be effective for recognition of *Aspergillus* species (12).

Triazoles and amphotericin B (AMB) are the drugs of choice for the management of human aspergillosis. Resistance to these drugs among *Aspergillus* species is an emerging public health concern worldwide (13, 14). In particular, reports of *A. flavus* being resistant to antifungal therapy and a case study from Brazil documented a patient who died of fungal sinusitis caused by a highly AMB resistant *Aspergillus* species (15). Antifungal susceptibility testing is significant to obtain insight into susceptibility profiles of the collected isolates and reveals drug resistance of clinical strains. In contrast to *A. fumigatus*, information regarding antifungal resistance mechanisms in *A. flavus* is relatively scarce (16). Recently, *A. flavus* isolates with *in vitro* azole resistance have been described and their resistance mechanisms are being analyzed (17).

The aim of our study is to investigate the prevalence, etiology, and resistance of FRS isolates from Sudan to elucidate the frequency of *A. flavus* in a dry tropical climate. In this study, data on etiologic agents of FRS were collected from the Mycology Reference Laboratory at the University of Khartoum, Sudan, covering the period from January 2015 to December 2019. Molecular identification was performed for a selected number of strains using three markers, that is, ITS, *CaM*, and *BenA*. Furthermore, antifungal susceptibility testing (AFST) was conducted for these strains using the protocol of the EUCAST. Finally, the *cyp51A* gene was sequenced to identify the mechanism of resistance in isolates with low susceptibility to antifungals.

## MATERIALS AND METHODS

### Prevalence of fungal rhinosinusitis

This study was a retrospective laboratory-based overview of 284 fungi causing rhinosinusitis from January 2015 to December 2019. All clinical strains were isolated previously from nasal biopsies of patients with FRS in Sudan and were identified by morphology at the Mycology Reference Laboratory at the University of Khartoum in Khartoum, Sudan. The isolates were preserved in 20% glycerol solutions at −80°C for further study. This research was a surveillance study and did not involve any human subjects.

### Phenotypic identification

One hundred clinical isolates were randomly selected from the above previous positive samples for morphological and molecular analysis. Suspensions of spores or mycelia were inoculated onto Sabouraud dextrose agar (SDA, Sigma Aldrich, Germany) plates and incubated in darkness at 37°C for 5–7 days as previously described (11). Microscopic mounting slides were prepared using lactic acid and examined by a light microscope (Zeiss Axio Scope A1, Carl Zeiss, Göttingen, Germany). Images were annotated in Adobe Photoshop 2022.

### Molecular identification

DNA extraction of the isolates grown on SDA for 3–7 days at 37℃ was performed as previously described (18).

The ITS rDNA region was amplified using primers ITS1 and ITS4, a part of the *BenA* gene encoding β-tubulin with primers β*tub1* and β*tub2* (19), and the partial *CaM* gene encoding calmodulin with primers cmd5 and cmd6 (20). PCR amplifications were performed as previously described (19). Gel Purification Kit (QIAquick, Hilden, Germany) was used for PCR product purification. Automated sequencing was performed with the same primers using Sanger Sequencing.

The sequencing results were assembled using SnapGene v6.0.2 and subjected to BLAST in the NCBI database (GenBank accession numbers provided in Table S1). Eighty-eight isolates belonging to the *A. flavus* complex were aligned by MAFFT v7 online (https://mafft.cbrc.jp/) and trimmed in BioEdit v7.2. The phylogenetic relationships were studied using concatenated sequences of *BenA*, and *CaM* sequences, analyzed with RAxML implemented in the CIPRES portal (https://www.phylo.org/). The maximum likelihood analysis included 1,000 bootstrap replicates. The resulting trees were visualized and annotated using ITOL online. Sequences of all type strains were downloaded from NCBI (Table S1), and *A. subflavus* CBS 14368^T^ was used as outgroup.

### *In vitro* antifungal susceptibility testing

The *in vitro* susceptibility of the isolates was evaluated against eight antifungals provided by Sigma-Aldrich (USA), including AMB, ravuconazole (RAV), caspofungin (CAS), micafungin (MCF), isavuconazole (ISA), itraconazole (ITZ), posaconazole (PCZ), and voriconazole (VCZ), using the broth microdilution method in accordance with the guidelines of the EUCAST. The drug concentration range for AMB, ITZ, VCZ, ISA, CAS, and RAV was 0.016–16 mg/L, for PCZ, it was 0.008–8 mg/L, and for MCF, it was 0.004–4 mg/L. The minimum effective concentration (MEC) for echinocandins was determined by observing abnormal, short, and branched hyphal clusters at the lowest drug concentrations, while for azoles and AMB, the minimum inhibitory concentration (MIC) was recorded as the lowest concentration at which no visible fungal growth was observed (EUCAST v9.4, effective from 1 April 2022). *Aspergillus fumigatus* ATCC 204305, *Candida parapsilosis* ATCC 22019, and *C. krusei* ATCC 6258 were used as quality controls. MIC/MEC data were interpreted according to the clinical breakpoints (BPs) or epidemiological cut-off values (ECOFFs) specified by EUCAST, if available. The MIC50 and MIC90 were calculated by arranging the MIC data of each antifungal in ascending order and selecting the median and 90th percentile values, respectively (21). Venn diagrams were generated using the online tool- Omicstudio (https://www.omicstudio.cn/).

### The *cyp51A* sequencing of triazole-resistant *A. flavus* and *A. fumigatus* isolates

The *cyp51A* gene (including the promoter region) for *A. flavus* and *A. fumigatus* non-WT strains were amplified and sequenced using the primers listed in Table S2 (19). Automated sequencing was performed with the same primers using Sanger Sequencer. The DNA sequences of *cyp51A* were aligned with those of the reference strains of *A. fumigatus* (GenBank AF338659) and *A. flavus* (GenBank XM002375082.1).

## RESULTS

### Prevalence and distribution

**Fig 1:**
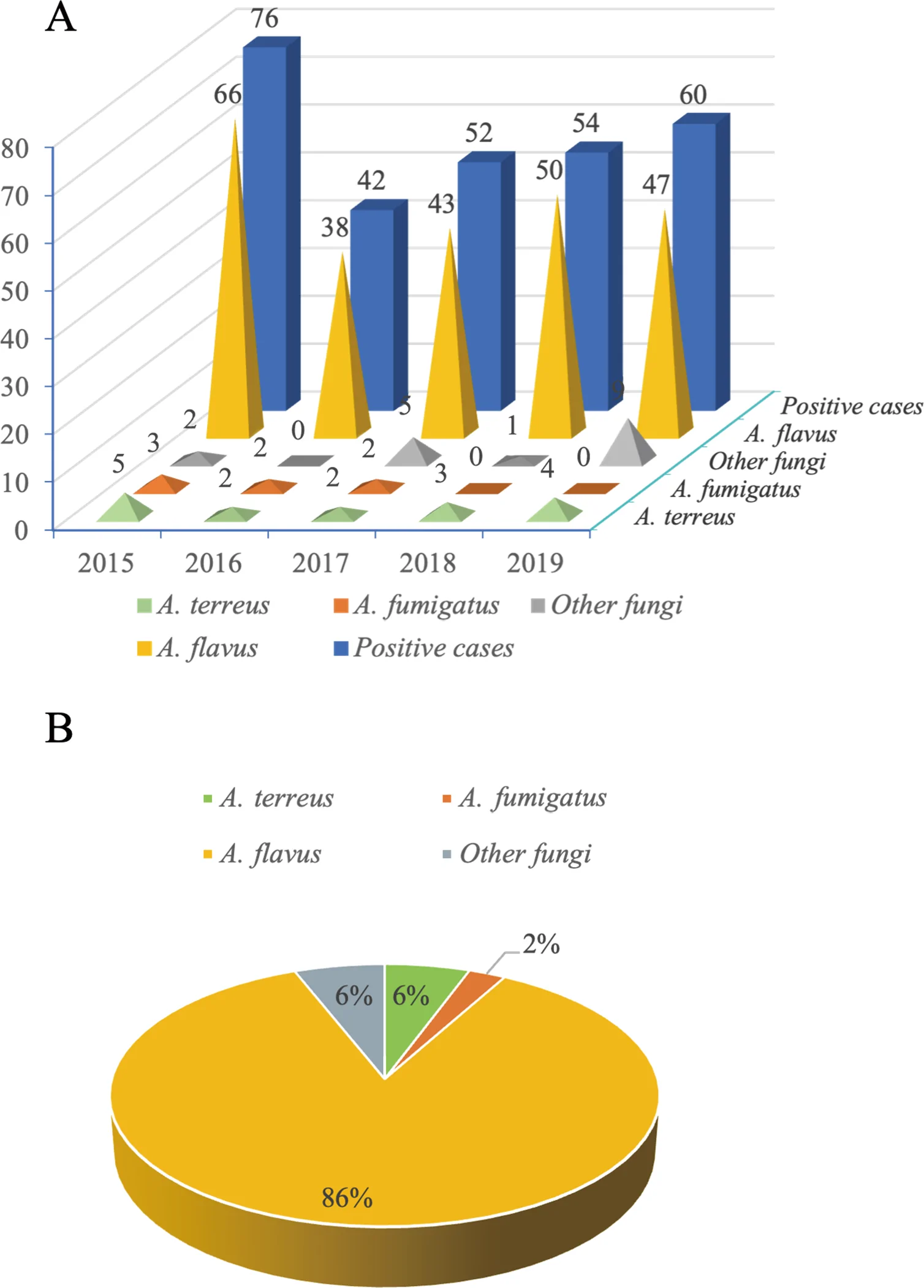
The frequency of FRS from 2015 to 2019 in Sudan. (A) The positive cases and pathogens. (B) The proportion of pathogens.

A total of 549 clinical samples were available during the study period. For every sample, histopathology, direct microscope, and culture were performed as the routine diagnostic procedures. Fungus-positive samples (i.e., with fungal hyphae in direct examination and growth in culture) were 52% (284/549) where the highest proportion was in 2015 and decreasing during subsequent years (Fig. 1A). Morphological identification of the cultures revealed members of the genus *Aspergillus* (*A. fumigatus*, *A. terreus*, and *A. flavus*) and occasionally other fungi (2 *Curvularia* sp., 2 *Bipolaris* sp., 1 *Candida* sp., and 12 unknown species; Fig. 1B). The genus *Aspergillus* was most commonly represented, accounting for 98% of all fungi causing FRS in this study. Furthermore, the *A. flavus* complex was the most frequent species group accounting for 86% (244/284). The *A. terreus* complex followed with 6% (16/284). The proportion of *A. fumigatus* was only 2% (7/284).

### Phenotypic analysis and molecular identification

**Fig 2:**
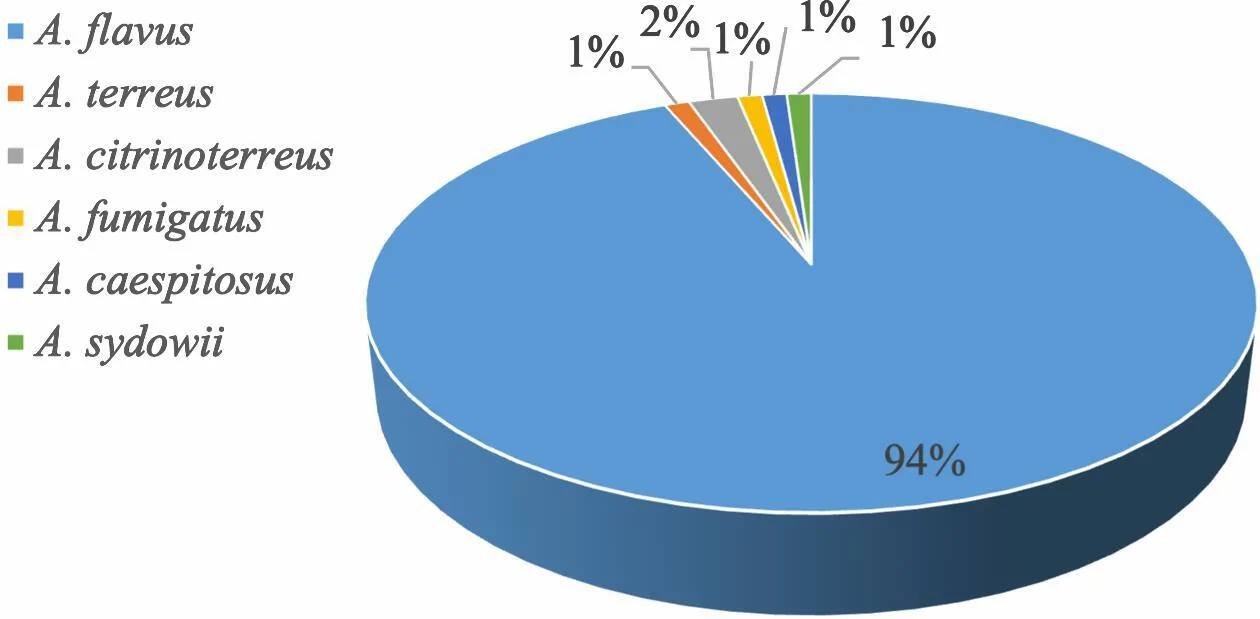
Proportion of isolates causing FRS identified using the molecular method.

Of the 100 selected isolates, we couldn't obtain good electropherogram for six isolates and 94 were used for further analysis. As shown in Fig. 2, the 94 sequenced *Aspergillus* strains belonged to six species including *A. flavus* (*n* = 88), *A. caespitosus* (*n* = 1), *A. sydowii* (*n* = 1), *A. citrinoterreus* (*n* = 2), *A. terreus* (*n* = 1), and *A. fumigatus* (*n* = 1). The six species of *Aspergillus* were distributed to four sections: eighty-eight isolates to section *Flavi*, one to section *Fumigati*, two to section *Nidulantes*, and three to section *Terrei*.

**Fig 3:**
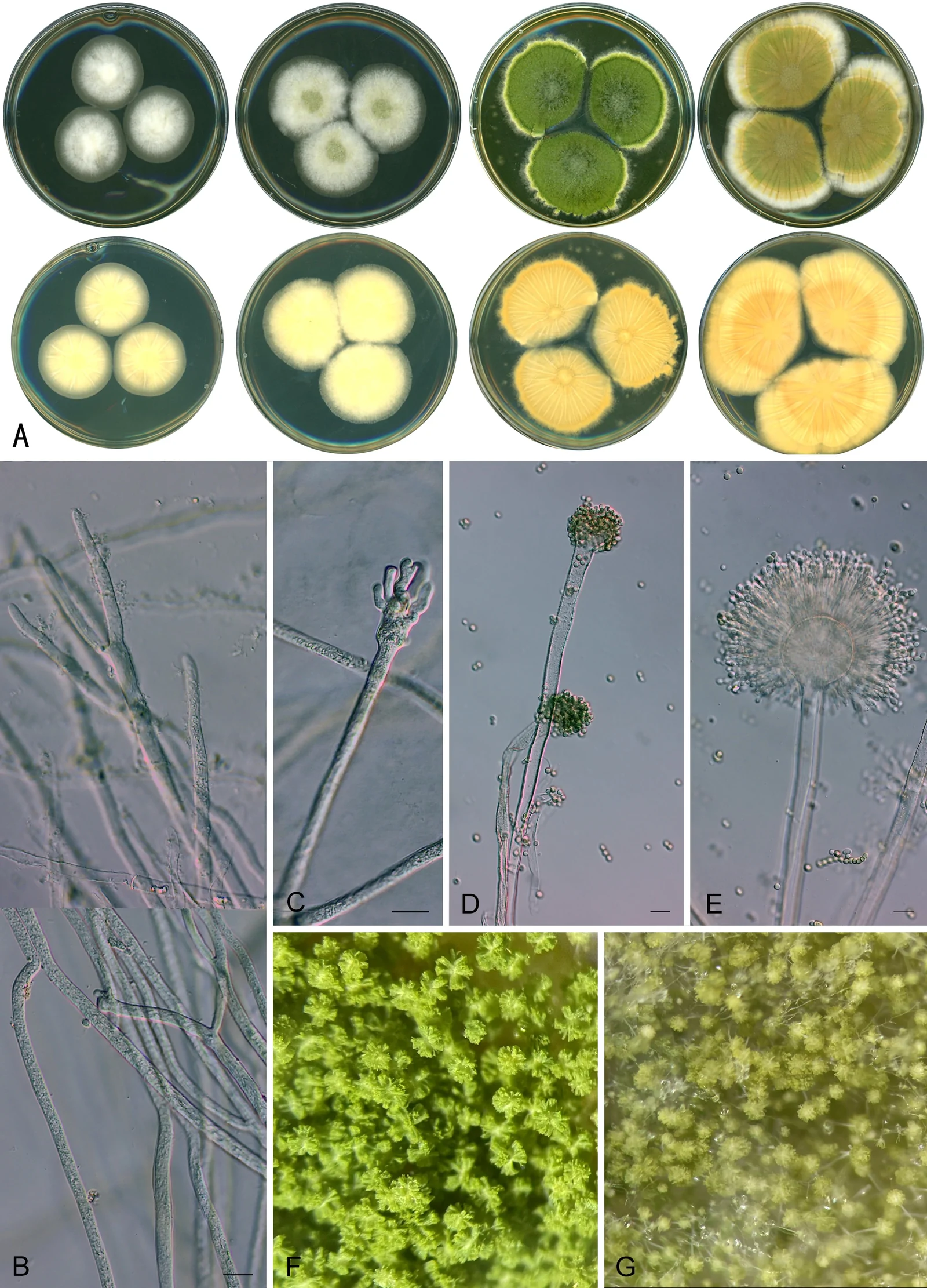
*Aspergillus flavus* phenotypes. (A) Colonies grown on SDA at 37°C for 5 days (from left to right: non-sporulation, sparse sporulation, yellowish-green conidia, and yellowish-brown conidia). (B) Non-sporulation. (C) Sparse sporulation. (D and F) Yellowish-green conidia. (E and G) Yellowish-brown conidia. Scale bar = 10 µM.

**Fig 4:**
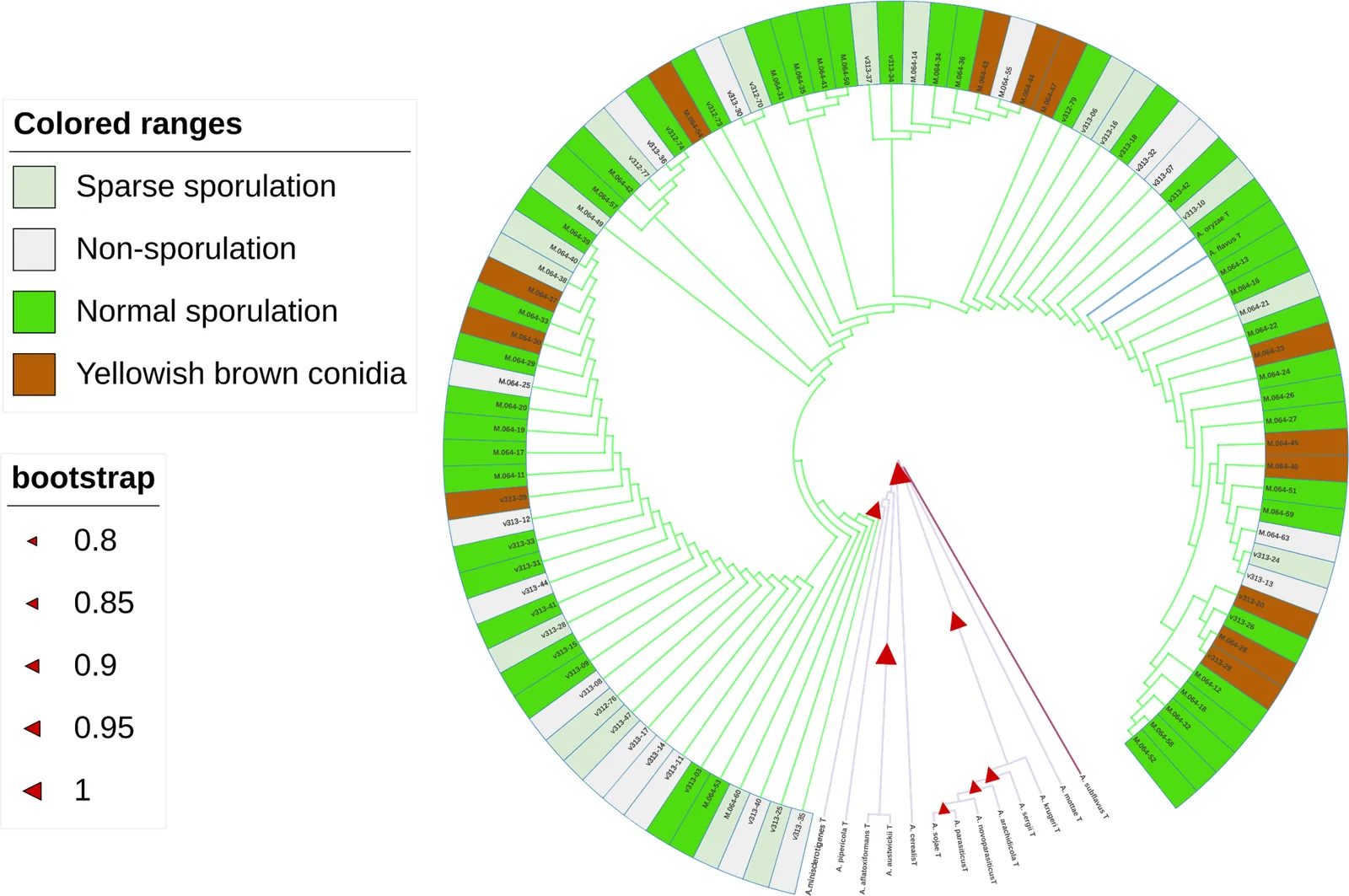
Sequence diversity inferred from a concatenated nucleotide data set (partial *BenA* and *CaM* sequences) using RAxML with 1,000 bootstrap replicates analysis showing the relationship of species accommodated in *A. flavus* clade of *Aspergillus* section *Flavi*. Bootstraps less than 0.8 were not shown in this tree. Names in black font represent strains causing FRS isolated in Sudan. Names with “T” together stand for type strains.

In section *Flavi*, four types of colony morphology on SDA could be distinguished (Fig. 3 and 4). Group 1 was degenerated and lacked sporulation (18.2%, 16/88). Group 2 exhibited sparse sporulation (19.3%, 17/88). Group 3 had spreading colonies with abundant sclerotia and yellow-green conidia (47.8%, 42/88). Group 4 had abundant sporulation with brownish conidia (14.7%, 13/88). Deviation from the standard *A. flavus* phenotype (=Group 3) was observed in 52% (46/88) of the isolates.

Sequence diversity of 88 *A*. *flavus* complex strains was studied using concatenated *BenA* and *CaM*. Fifteen type strains of the *A. flavus* complex were included for comparison. The total length of the aligned data set was 960 characters, containing 460 and 500bp for *BenA* and *CaM*, respectively. All 88 isolates belonged to the *A. flavus/A. oryzae* sibling (Fig. 4), albeit clustering into several unsupported subclades. The type strains of *A. flavus* (CBS 569.65) and *A. oryzae* (CBS 102.07) are identical with these two markers, and thus, the clade was referred to as *A. flavus/A. oryzae*. Since *A. oryzae* is nonclinical, our strains are identified here as *A. flavus*. The phenotypes of the isolates (non-sporulation, sporulation sparse, and yellowish-brown conidia) could not be correlated with genotypes/subclades.

### *In vitro* antifungal susceptibility

The activities of eight antifungal agents were tested against 94 *Aspergillus* strains (Table 1) with reference to the current EUCAST ECOFFs (WT, mg/L) and susceptibility breakpoints (S, mg/L) for *Aspergillus* (22). In *A. flavus*, 98.9%, 97.7%, 96.6%, 97.7%, and 93.3% were susceptible or WT to AMB, ITZ, VCZ, PCZ, and ISA, respectively. The MIC/MEC50 and MIC/MEC90 of AMB, ITZ, VCZ, PCZ, ISA, CAS, MCF, and RAV were 2/4, 0.25/0.5, 1/2, 0.25/0.25, 2/2, 0.25/0.5, 0.062/0.125, and 1/2 mg/L, respectively. Of the total *A. flavus* isolates, 11.4% (*n* = 10) had MICs of ≥4 mg/L and 58.0% (*n* = 51) ≥2 mg/L to AMB (Table 1). Concerning the triazoles, 97.7% (*n* = 86) of the isolates had MICs of ≤0.5 mg/L to ITZ and PCZ. Thirty isolates showed MICs of ≥2 mg/L to RAV. The MEC50/90 of *A. flavus* against CAS and MCF were 0.25/0.5 and 0.06/0.125, respectively.

**TABLE 1:** Antifungal susceptibility and MIC/MEC distributions for 94 *Aspergillus isolates* causing FRS^*a*^

Species	Antifungal agents		No. of isolates												MIC/MEC (mg/L)			EUCAST (breakpoint) (mg/L)		ECOFF (mg/L)	
		MIC/MEC (mg/L) range	0.008	0.016	0.031	0.062	0.125	0.25	0.5	1	2	4	8	16	GM	MIC50	MIC90	S (*n*, %)	R (*n*, %)	WT (*n*, %)	Non-WT (*n*, %)
*A. flavus complex* (*n* = 88)	AMB	0.25–8						1	4	32	41	9	1		1.55	2	4	NB	NB	87 (98.9%)	1 (1.2%)
	ITZ	0.062–16				2	10	57	17		1			1	0.27	0.25	0.5	86(97.7%)	2 (2.3%)	86 (97.7%)	2 (2.3%)
	VCZ	0.25–4						1	10	45	29	3			1.2	1	2	ND	ND	85 (96.6%)	3 (3.4%)
	PCZ	0.008–8	1			3	32	46	4	1			1		0.19	0.25	0.25	ND	ND	86 (97.7%)	2 (2.3%)
	ISA	0.25–8						1	5	34	44	3	1		1.44	2	2	82(93.3%)	6 (6.8%)	84 (95.5%)	4 (4.5%)
	CAS	0.125–0.5					22	47	19						0.24	0.25	0.5	NB	NB	NB	NB
	MCF	0.008–0.25	2	8	19	35	21	3							0.06	0.062	0.125	NB	NB	NB	NB
	RAV	0.25–8						1	5	34	44	3	1		1.08	1	2	NB	NB	NB	NB
*A. terreus* (*n* = 3)	AMB	1–4								1		2			2.52	NA	NA	NB	NB	3 (100%）	0
	ITZ	0.062–0.125				2	1								0.08	NA	NA	3(100%）	0	3(100%）	0
	VCZ	1–2								2	1				1.26	NA	NA	NB	NB	3(100%）	0
	PCZ	0.031–0.062			2	1									0.04	NA	NA	3(100%）	0	3(100%）	0
	ISA	1–2								2	1				1.26	NA	NA	3(100%）	NB	2(66.7%)	1(33.3%）
	CAS	0.016–2		1							2				0.4	NA	NA	NB	NB	NB	NB
	MCF	0.016–0.125		1		1	1								0.05	NA	NA	NB	NB	NB	NB
	RAV	0.5–1							2	1					0.63	NA	NA	NB	NB	NB	NB
*A. nidulans* (*n* = 2)	AMB	0.5–4							1			1			1.41	NA	NA	NB	NB	2(100%）	0
	ITZ	0.031–1			1					1					0.18	NA	NA	2(100%）	0	2(100%）	0
	VCZ	0.25–1						1		1					0.5	NA	NA	NB	NB	2(100%）	0
	PCZ	0.031–0.5		1					1						0.09	NA	NA	2(100%）	0	2(100%）	0
	ISA	0.5–2							1		1				1	NA	NA	0	2(100%）	0	2(100%）
	CAS	0.062–1				1				1					0.25	NA	NA	NB	NB	NB	NB
	MCF	0.008–0.5	1						1						0.06	NA	NA	NB	NB	NB	NB
	RAV	0.25–1						1		1					0.5	NA	NA	NB	NB	NB	NB
*A. fumigatus* (*n* = 1)	AMB	0.5							1						0.5	NA	NA	1(100%）	0	1(100%）	0
	ITZ	>16												1	>16	NA	NA	0	1(100%）	0	1(100%）
	VCZ	4										1			4	NA	NA	0	1(100%）	0	1(100%）
	PCZ	1								1					1	NA	NA	0	1(100%）	0	1(100%）
	ISA	8											1		8	NA	NA	0	1(100%）	0	1(100%）
	CAS	0.25						1							0.25	NA	NA	NB	NB	NB	NB
	MCF	0.062			1										0.062	NA	NA	NB	NB	NB	NB
	RAV	4										1			4	NA	NA	NB	NB	NB	NB

^*a*^ AMB, amphotericin B; ITZ, itraconazole; VCZ, voriconazole; PCZ, posaconazole; ISA, isavuconazole; RAV, ravuconazole; CAS, caspofungin; MCF, micafungin; S, susceptible; GM, geometric mean; MEC, minimal effective concentration; MIC, minimum inhibitory concentration; R, resistant; WT, wild type; NB, no breakpoint; NA, not available (MIC/MEC50/90 calculated from ≥10 isolates).

**Fig 5:**
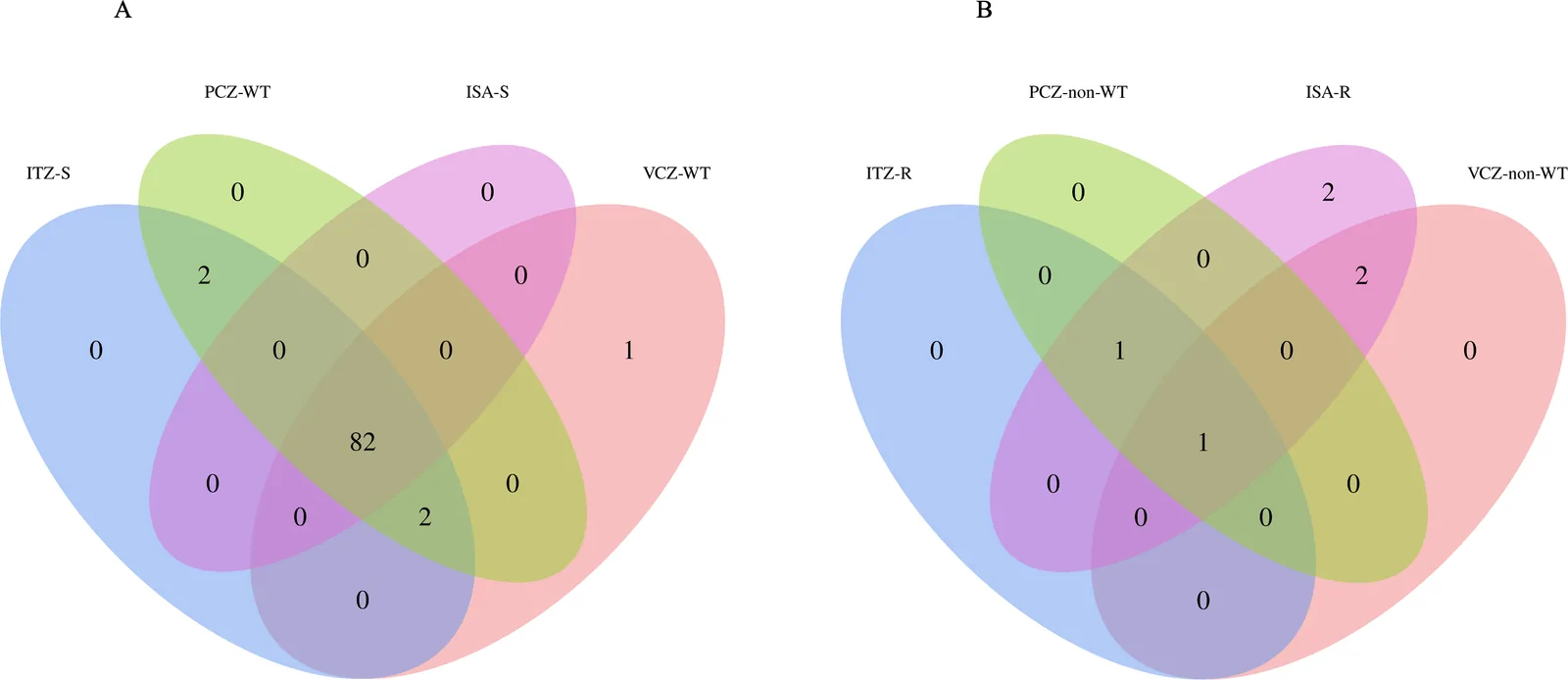
Venn diagram of antifungal susceptibility to azoles for *A. flavus isolates*. (A) WT/susceptible isolates. (B) Non-WT/resistant isolates. ITZ, itraconazole; VCZ, voriconazole; PCZ, posaconazole; ISA, isavuconazole; S, susceptible; R, resistant; WT, wild type.

The susceptibility profiles of *A. terreus* complex (*A. terreus* and *A. citrinoterreus*) (*n* = 3), *A. nidulans* complex (*A. caespitosus* and *A. sydowii*) (*n* = 2), and *A. fumigatus* (*n* = 1) isolates were listed (Table 1). A Venn diagram (Fig. 5) was constructed to display that 93.3% (*n* = 82) of the isolates of *A. flavus* shared a susceptible phenotype or WT to the four azoles (ITZ, VCZ, PCZ, and ISA), while 97.7% (*n* = 86) strains shared susceptibility or WT to ITZ and PCZ. Cross-resistance or non-WT to ITZ, VCZ, PCZ, and ISA was observed in three isolates sharing a non-WT or resistant phenotype to VCZ and ISA, two isolates shared resistance to ITZ and PCZ, and one isolate displayed multi-resistance to all triazoles.

### Detection of *cyp51A* single nucleotide polymorphisms (SNP) in azole-resistant isolates

Four *A. flavus* isolates with low susceptibility to azoles (shown in Table S3) were identified, and their genomic sequences were analyzed. These isolates had synonymous SNPs in the *cyp51A* coding regions, including P55P, K130K, F182F, and P388P. Additionally, M.064–11 and M.064–12 had other mutations in their sequences, including H355H, N456N, L457L, and A199T in M.064–12. The *A. fumigatus*-resistant strain had four non-synonymous SNPs (F46Y, M172V, N248T, and E427K) and three synonymous SNPs (G98G, L358L, and C454C) in its *cyp51A* coding regions. Table S3 provides more information about these mutations.

## DISCUSSION

This study presented an overview of the prevalence of *Aspergillus* from FRS in an arid climate region of Sudan. The 5-year data, from 2015 to 2019, showed that *Aspergillus* species were the prevalent fungi accounting for 98% of the fungus-positive cases, with *A. flavus* complex (244/284, 86%) as the predominant species. *Aspergillus flavus* prevalence observed in our study has slightly increased compared with that in an earlier report from Sudan covering the years 2010 to 2015 (5), where *A. flavus* accounted for 77%. Prevalence was also higher than that observed in other arid or tropical regions, in several epidemiological studies, which listed 58.8% (10/17) and 64.2% (52/81) in Saudi Arabia and India, respectively (23, 24). On the other hand, Comacle et al. (25) found *A. fumigatus* as the main etiologic agent (69%) of FRS in temperate conditions in France, while *A. flavus* was responsible for only 7% of the cases. The species differ in clinical spectrum and distribution: *A. fumigatus* is the prevalent species causing invasive aspergillosis worldwide, while *A. flavus* is predominantly reported from tropical regions causing a wide spectrum of clinical conditions (1). The differences in distribution and clinical spectra of these two species are likely related to their ecological preferences and the specific virulence factors they produce. In addition, it has been suggested that *A. flavus* was prevalent in sinusitis due to its larger conidium size (3–6 µm), against 2–3.5 µm in *A. fumigatus* (26). Furthermore, remarkable differences in the conidial cell wall structure were found (27).

An interesting phenomenon regarding our *A. flavus* isolates was their morphological instability, which had been shown in previous studies of strains from pulmonary aspergillosis that displayed high diversity, possibly in response to the host’s stressful conditions (28). Low nutrient availability, hypoxia, and antifungal treatment, in the host, can affect the growth rate and conidiation capacity of *A. flavus*. Strains lacking sporulating are difficult to identify in the routine laboratory, and additional media are required to promote a recognizable phenotype (29). The presence of different phenotypes was not observed in *A. fumigatus* but was previously reported for *A. flavus*, which has important implications for host immunological reactivities (27). Sequencing data of *BenA* and *CaM* confirmed that all our 88 isolates were clustered in one clade with the type strains of *A. flavus* and *A. oryzae*, indicating that a single species is involved (30, 31).

Our observation of an overwhelming prevalence of *A. flavus* isolates in comparison with other species in section *Flavi* is in line with what had been observed by Al-Wathiqi et al. (32) who identified 92 clinical and 7 environmental isolates, all being *A. flavus*. The determining factors that set the species apart from its siblings in clinical settings are unknown. The species has a worldwide distribution, producing large amounts of easily dispersed conidia, is thermotolerant, and is possibly more resistant to antifungal drugs than other species in section *Flavi*. These characteristics make it more likely to colonize cavities like sinuses and harder to treat once an infection has occurred (31). Additionally, the immune status of the host may also play a role, with previous cases in Sudan being immunocompetent patients (5). Our study has limitations here as clinical and immunological profiles of the patients were not available and further research is needed to investigate this in more depth.

This study revealed for the first time the presence of some cryptic species, including *A. caespitosus* (1/94) and *A. sydowii* (1/94) in the section *Nidulantes* causing FRS in Sudan. *Aspergillus caespitosus* is a soil fungus (33) and has been reported previously from BAL specimen of a patient suffering from tuberculosis in Qatar (34). *Aspergillus sydowii* was reported in patients with otomycosis in Brazil (35).

Several of our *A. flavus* isolates exhibited low susceptibility to azoles, with 3.4% of isolates having higher MICs to VCZ than what has been reported in China (1.2%) (36). With respect to ISA, this antifungal agent has been shown to be active *in vitro* against several species of *Aspergillus* and *Candida* (37). However, our result showed that 4.5% of *A. flavus* isolates were non-WT to ISA, similar to data (4.2%) reported by Pfaller et al. (38). The elevated MIC50 and MIC90 (2 and 2 mg/L) against ISA in our study were also comparable with previous findings on clinical and environmental isolates of *A. flavus* (1 µg/mL) from Spain (39). It is noteworthy that in Sudan, only ITZ is available for treating FRS and there has been no reports of resistance to this drug (5). However, our results for ITZ and PCZ showed that 2.3% of *A. flavus* isolates had MICs above EUCAST breakpoints and ECOFFs. To investigate the genetic basis of azole resistance, we sequenced the *cyp51A* gene of four *A. flavus* and one *A. fumigatus* with decreased azole susceptibility. The four *A. flavus* isolates shared the same SNPs in the *cyp51A* gene, which have also been reported by Rivero-Menendez et al. (40). However, it is unclear whether these SNPs contribute to azole resistance, as transformation experiments have not been conducted. Although a novel Y119F substitution in the *cyp51A* gene is promising as a key player in azole resistance (28), we did not encounter this mutation and no obvious resistance-associated mutations were identified. Additional work is needed to find potential SNPs that could explain the increased MICs in *A. flavus*. In addition, a correlation between the high MICs and clinical outcome needs to be established. One *A. fumigatus* had some SNPs, which have been found in both azole-resistant and susceptible isolates (41, 42).

Regarding AMB, our results showed lower MICs and only 1.2% of the isolates were non-WT. Other studies have reported higher resistance rates (14.3% in Europe, 11.7% in USA) to AMB in *A. flavus* (43). Lass-Floerl et al. (44) found that 67% of *A. flavus* isolates in Austria had high MIC to AMB and were associated with AMB therapy failure. These reports suggest intrinsic resistance to this drug in this species. For echinocandins, both CAS and MCF demonstrated excellent *in vitro* activity against all *A. flavus* isolates tested in our study, with lower MECs compared with triazoles and AMB. This finding is consistent with previous reports that have also demonstrated the effectiveness of echinocandins against *A. flavus* (45).

### Conclusions

*Aspergillus* was the most common fungus associated with rhinosinusitis in Sudan, and molecular identification is useful to identify cryptic species and non-sporulating *A. flavus* isolates. The *Aspergillus* isolates studied were found to belong to six species distributed in four sections of the genus *Aspergillus* (*Flavi*, *Terrei*, *Nidulantes*, and *Fumigati*). As the predominant species causing FRS in Sudan is *A. flavus*, emerging azole resistance of 2.3%–4.5% is a public health concern. Our results underscore the importance of monitoring antifungal susceptibility in *A. flavus* isolates to guide treatment decisions and ensure efficacy, particularly in regions where only limited antifungal options are available.

## SUPPLEMENTAL MATERIAL

Supplemental tables spectrum.01831-23-s0001.docx Tables S1 to S3.

ASM does not own the copyrights to Supplemental Material that may be linked to, or accessed through, an article. The authors have granted ASM a non-exclusive, world-wide license to publish the Supplemental Material files. Please contact the corresponding author directly for reuse.
