# Supplementary material for: Fungi involved in rhinosinusitis in arid regions: insights from molecular identification and antifungal susceptibility
Source: Microbiol Spectr. 2023 Sep 29;11(5):e01831-23. doi: 10.1128/spectrum.01831-23 (PMC10580872; doi:10.1128/spectrum.01831-23)
Supplement: Supplemental tables — Tables S1 to S3. [file spectrum.01831-23-s0001.docx]

Table S1 Information of strains used in our study

|  |  |  |  |  |  |
| --- | --- | --- | --- | --- | --- |
| Species name | | Collection no. | GenBank accession nr. | | |
|  |  |  | *BenA* | *CaM* | ITS |
| *A. oryzae ^T^* | | CBS 102.07 ^T^ | EF661483.1 | EF661506.1 | NR135395 |
| *A. flavus ^T^* | | CBS 569.65 ^T^ | EF661485.1 | EF661508.1 | AF027863.1 |
| *A. cerealis ^T^* | | CBS 143674 ^T^ | MG517693.1 | MG518063.1 | MG662394.1 |
| *A. austwickii ^T^* | | CBS 143677 ^T^ | MG517702.1 | MG518072.1 | MG662391.1 |
| *A. aflatoxiformans ^T^* | | CBS 143679 ^T^ | MG517706.1 | MG518076.1 |  |
| *A. pipericola ^T^* | | CBS 143680 ^T^ | MG517717.1 | MG518087.1 | MG662385.1 |
| *A. minisclerotigenes ^T^* | | CBS 117635 ^T^ | EF203148.1 | MG518009.1 | EF409239.1 |
| *A. sojae ^T^* | | CBS 100928 ^T^ | EF203168.1 | EF202041.1 | KJ175434.1 |
| *A. parasiticus ^T^* | | CBS 100926 ^T^ | EF661481.1 | EF661516.1 | AF027862.1 |
| *A. arachidicola ^T^* | | CBS 117610 ^T^ | EF203158.1 | EF202049.1 | MF668184.1 |
| *A. novoparasiticus ^T^* | | CBS 126849 ^T^ | MG517684.1 | MG518055.1 | MG662397.1 |
| *A. sergii ^T^* | | CBS 130017 ^T^ | MG517688.1 | MG518059.1 | JF412769.1 |
| *A. krugeri ^T^* | | PPRI8986 ^T^ | MK451098.1 | MK451517.1 | - |
| *A. mottae ^T^* | | CBS 130016 ^T^ | MG517687.1 | MG518058.1 | F412767.1 |
| *A. subflavus ^T^* | | CBS 143683 ^T^ | MG517773.1 | MG518143.1 | MH279429.1 |
| *A.caespitosus* | | v313-04 | OR451368 | OR451274 | OR418500 |
| 1. *sydowii* | | v313-23 | OR451369 | OR451275 | OR418541 |
| *A. terreus* | | M.064-61 | OR451370 | OR451276 |  |
| *A. citrinoterreus* | | v312-75 | OR451371 | OR451277 | OR418542 |
| *A. citrinoterreus* | | v313-05 | OR451372 | OR451278 | OR418543 |
| *A. fumigatus* | | M.064-62 | OR451373 | OR451279 |  |
| *A. flavus* | | v312-70 | OR451280 | OR451374 | OR418501 |
| *A. flavus* | | v312-73 | OR451281 | OR451375 | OR418502 |
| *A. flavus* | | v312-74 | OR451282 | OR451376 | OR418503 |
| *A. flavus* | | v312-76 | OR451283 | OR451377 | OR418504 |
| *A. flavus* | | v312-77 | OR451284 | OR451378 | OR418505 |
| *A. flavus* | | v312-79 | OR451285 | OR451379 | OR418506 |
| *A. flavus* | | v313-03 | OR451286 | OR451380 | OR418507 |
| *A. flavus* | | v313-06 | OR451287 | OR451381 | OR418508 |
| *A. flavus* | | v313-07 | OR451288 | OR451382 | OR418509 |
| *A. flavus* | | v313-08 | OR451289 | OR451383 | OR418510 |
| *A. flavus* | | v313-09 | OR451290 | OR451384 | OR418511 |
| *A. flavus* | | v313-10 | OR451291 | OR451385 | OR418512 |
| *A. flavus* | | v313-11 | OR451292 | OR451386 | OR418513 |
| *A. flavus* | | v313-12 | OR451293 | OR451387 | OR418514 |
| *A. flavus* | | v313-13 | OR451294 | OR451388 | OR418515 |
| *A. flavus* | | v313-14 | OR451295 | OR451389 | OR418516 |
| *A. flavus* | | v313-15 | OR451296 | OR451390 | OR418517 |
| *A. flavus* | | v313-16 | OR451297 | OR451391 | OR418518 |
| *A. flavus* | | v313-17 | OR451298 | OR451392 | OR418519 |
| *A. flavus* | | v313-18 | OR451299 | OR451393 | OR418520 |
| *A. flavus* | | v313-20 | OR451300 | OR451394 | OR418521 |
| *A. flavus* | | v313-24 | OR451301 | OR451395 | OR418522 |
| *A. flavus* | | v313-25 | OR451302 | OR451396 | OR418523 |
| *A. flavus* | | v313-26 | OR451303 | OR451397 | OR418524 |
| *A. flavus* | | v313-28 | OR451304 | OR451398 | OR418525 |
| *A. flavus* | | v313-29 | OR451305 | OR451399 | OR418526 |
| *A. flavus* | | v313-30 | OR451306 | OR451400 | OR418527 |
| *A. flavus* | | v313-31 | OR451307 | OR451401 | OR418528 |
| *A. flavus* | | v313-32 | OR451308 | OR451402 | OR418529 |
| *A. flavus* | | v313-33 | OR451309 | OR451403 | OR418530 |
| *A. flavus* | | v313-34 | OR451310 | OR451404 | OR418531 |
| *A. flavus* | | v313-35 | OR451311 | OR451405 | OR418532 |
| *A. flavus* | | v313-36 | OR451312 | OR451406 | OR418533 |
| *A. flavus* | | v313-37 | OR451313 | OR451407 | OR418534 |
| *A. flavus* | | v313-39 | OR451314 | OR451408 | OR418535 |
| *A. flavus* | | v313-40 | OR451315 | OR451409 | OR418536 |
| *A. flavus* | | v313-41 | OR451316 | OR451410 | OR418537 |
| *A. flavus* | | v313-42 | OR451317 | OR451411 | OR418538 |
| *A. flavus* | | v313-44 | OR451318 | OR451412 | OR418539 |
| *A. flavus* | | v313-47 | OR451319 | OR451413 | OR418540 |
| *A. flavus* | | M.064-11 | OR451320 | OR451414 |  |
| *A. flavus* | | M.064-12 | OR451321 | OR451415 |  |
| *A. flavus* | | M.064-13 | OR451322 | OR451416 |  |
| *A. flavus* | | M.064-14 | OR451323 | OR451417 |  |
| *A. flavus* | | M.064-16 | OR451324 | OR451418 |  |
| *A. flavus* | | M.064-17 | OR451325 | OR451419 |  |
| *A. flavus* | | M.064-18 | OR451326 | OR451420 |  |
| *A. flavus* | | M.064-19 | OR451327 | OR451421 |  |
| *A. flavus* | | M.064-20 | OR451328 | OR451422 |  |
| *A. flavus* | | M.064-21 | OR451329 | OR451423 |  |
| *A. flavus* | | M.064-22 | OR451330 | OR451424 |  |
| *A. flavus* | | M.064-23 | OR451331 | OR451425 |  |
| *A. flavus* | | M.064-24 | OR451332 | OR451426 |  |
| *A. flavus* | | M.064-25 | OR451333 | OR451427 |  |
| *A. flavus* | | M.064-26 | OR451334 | OR451428 |  |
| *A. flavus* | | M.064-27 | OR451335 | OR451429 |  |
| *A. flavus* | | M.064-28 | OR451336 | OR451430 |  |
| *A. flavus* | | M.064-29 | OR451337 | OR451431 |  |
| *A. flavus* | | M.064-30 | OR451338 | OR451432 |  |
| *A. flavus* | | M.064-31 | OR451339 | OR451433 |  |
| *A. flavus* | | M.064-32 | OR451340 | OR451434 |  |
| *A. flavus* | | M.064-33 | OR451341 | OR451435 |  |
| *A. flavus* | | M.064-34 | OR451342 | OR451436 |  |
| *A. flavus* | | M.064-35 | OR451343 | OR451437 |  |
| *A. flavus* | | M.064-36 | OR451344 | OR451438 |  |
| *A. flavus* | | M.064-37 | OR451345 | OR451439 |  |
| *A. flavus* | | M.064-38 | OR451346 | OR451440 |  |
| *A. flavus* | | M.064-39 | OR451347 | OR451441 |  |
| *A. flavus* | | M.064-40 | OR451348 | OR451442 |  |
| *A. flavus* | | M.064-41 | OR451349 | OR451443 |  |
| *A. flavus* | | M.064-42 | OR451350 | OR451444 |  |
| *A. flavus* | | M.064-43 | OR451351 | OR451445 |  |
| *A. flavus* | | M.064-44 | OR451352 | OR451446 |  |
| *A. flavus* | | M.064-45 | OR451353 | OR451447 |  |
| *A. flavus* | | M.064-46 | OR451354 | OR451448 |  |
| *A. flavus* | | M.064-47 | OR451355 | OR451449 |  |
| *A. flavus* | | M.064-49 | OR451356 | OR451450 |  |
| *A. flavus* | | M.064-50 | OR451357 | OR451451 |  |
| *A. flavus* | | M.064-51 | OR451358 | OR451452 |  |
| *A. flavus* | | M.064-52 | OR451359 | OR451453 |  |
| *A. flavus* | | M.064-53 | OR451360 | OR451454 |  |
| *A. flavus* | | M.064-54 | OR451361 | OR451455 |  |
| *A. flavus* | | M.064-55 | OR451362 | OR451456 |  |
| *A. flavus* | | M.064-57 | OR451363 | OR451457 |  |
| *A. flavus* | | M.064-58 | OR451364 | OR451458 |  |
| *A. flavus* | | M.064-59 | OR451365 | OR451459 |  |
| *A. flavus* | | M.064-60 | OR451366 | OR451460 |  |
| *A. flavus* | | M.064-63 | OR451367 | OR451461 |  |

Table S2 The *cyp51A* primers of *A. flavus* and *A. fumigatus* (19)

| Primer name | Primer sequence (5’–3’) | Position on coordinate (bases) | Species |
| --- | --- | --- | --- |
| AflaCYP51A F1 | CAAGAACAGCCTGCACAGAG | 324 | *A. flavus* |
| AflaCYP51AR1 | GGGTGGATCAGTCTTATTA | 1126 |  |
| AflaCYP51AF2 | GCAATCATCGTCCTAAATC | 1066 |  |
| AflaCYP51AR2 | CTGTCCATTCTTGTAGGTA | 1899 |  |
| AflaCYP51AF3 | GCATGAGGGAGATCTATATG | 1791 |  |
| AflaCYP51AR3 | CCTATAATTGCTGGTTTCG | 2649 |  |
| AflaCYP51AF4 | TGAAGCTATTCAATGTAGAC | 2480 |  |
| AflaCYP51AR4 | ACTGCTGATGGTGTGCTAAG | 3358 |  |
| A205T-F | GGAGTCGCATGTACCATTGA | 1510 |  |
| A205T-R | TGAAGTTGATCGGAGTGAACC | 1716 |  |
| Forward | 5’-ATGGTGCCGATGCTATGG-3’ |  | *A. fumigatus* |
| Reverse | 5’-CTGTCTCACTTGGATGTG-3’ |  |  |

Table S3 Mutations found in the *cyp51A* gene of low susceptible strains and azole susceptibility phenotypes

| Taxonomy | Strain No. | GenBank accession nr. | MIC (mg/L) | | | | Amino acid change in *cyp51A* |
| --- | --- | --- | --- | --- | --- | --- | --- |
|  |  | CYP51A | ITZ | VCZ | PCZ | ISA |  |
| *A. fumigatus* | M.064-62 | OR454496 | >16 | 4 | 1 | 8 | F46Y, G89, M172V, N248T, D255D, V288I, L358L, E427K, C454C |
| *A. flavus* | v313-29 | OR456145 | 2 | 2 | 1 | 8 | P55P, K130K, F182F, P388P |
|  | M.064-11 | OR456142 | 0.25 | 4 | 0.125 | 2 | P55P, K130K, F182F, H355H, P388P, N456N, L457L |
|  | M.064-12 | OR456143 | 0.25 | 4 | 0.25 | 2 | P55P, K130K, F182F, A199T, H355H, P388P, N456N, L457L |
|  | M.064-28 | OR456144 | 16 | 4 | 8 | 4 | P55P, K130K, F182F, P388P |
